# LPS-induced extracellular AREG triggers macrophage pyroptosis through the EGFR/TLR4 signaling pathway

**DOI:** 10.3389/fimmu.2025.1549749

**Published:** 2025-04-11

**Authors:** Gang Yuan, Qudi Qiao, Aolin Jiang, Zehui Jiang, Haihua Luo, Lin Huang, Jieyan Wang, Yong Jiang

**Affiliations:** ^1^ Guangdong Provincial Key Laboratory of Proteomics, School of Basic Medical Sciences, Southern Medical University, Guangzhou, Guangdong, China; ^2^ Department of Critical Care Medicine, The Third Affiliated Hospital of Southern Medical University, Guangzhou, Guangdong, China; ^3^ Department of Urology, People’s Hospital of Longhua, Shenzhen, Guangdong, China; ^4^ Department of Respiratory and Critical Care Medicine, The Tenth Affiliated Hospital (Dongguan People’s Hospital), Southern Medical University, Dongguan, Guangdong, China; ^5^ Henan International Joint Laboratory of Infection and lmmmunology, The First Affiliated Hospital, Zhengzhou University, Zhengzhou, Henan, China; ^6^ Henan Key Laboratory of Critical Care Medicine, Department of Emergency Medicine, The First Affiliated Hospital, Zhengzhou University, Zhengzhou, Henan, China; ^7^ Institute of Infection and Immunity, Henan Academy of Innovations in Medical Science, Zhengzhou, Henan, China

**Keywords:** amphiregulin, EGFR/TLR4, macrophage, pyroptosis, sepsis

## Abstract

Amphiregulin (AREG), a member of the EGF family, exists as a transmembrane protein anchored to the cell surface. In response to external stimuli, its extracellular domain is released into the extracellular matrix through paracrine or autocrine signaling. However, its role in septic macrophage pyroptosis remains poorly understood. This study aims to investigate the role of extracellular AREG in septic macrophages, mice, and patients. We found that high expression of extracellular AREG was regulated by RPLP1 at the translation level, which increased the expression of IL-6, CCL2, and CCL3 protein, as well as *Caspase 1, IL-1β*, and *Nlrp3* mRNA expression, resulting in macrophage pyroptosis. Mechanistically, macrophage pyroptosis was aggravated by extracellular AREG pretreatment, which was triggered by extracellular AREG and ATP (adenosine 5′-triphosphate). The AREG-neutralizing antibody reduced LPS-induced epidermal growth factor receptor (EGFR) activation, TLR4 expression, and pyroptosis. Extracellular AREG-induced macrophage pyroptosis decreased with EGFR and NF-κB inhibition, as well as TLR4 and Myd88 knockout. Additionally, DTT-pretreated extracellular AREG suppressed macrophage pyroptosis. *In vivo*, extracellular AREG attenuates systemic inflammation infiltration and delays survival in a septic mouse model. Furthermore, extracellular AREG mediates sepsis in humans, and genes involved in the AREG-mediated pyroptosis signaling pathway were highly expressed in patients with severe sepsis compared with those with general or moderate sepsis. Overall, LPS-induced extracellular AREG aggravated or triggered macrophage pyroptosis through the EGFR/TLR4/Myd88/NF-κB signaling pathway, providing promising treatment strategies for sepsis.

## 1 Introduction

Sepsis is a life-threatening pathophysiological response to infection characterized by excessive inflammation and immunosuppression (1). Sepsis remains one of the most common infections in patients who are critically ill, resulting in high morbidity, mortality, and treatment costs (2). Therefore, elucidating the cellular and molecular mechanisms underlying this phenomenon is critical. After invading the body, pathogens bind to pattern recognition receptors on macrophages, triggering the secretion of pro-inflammatory cytokines (3). These cytokines increase the activation of immune cells through autocrine and paracrine pathways, disrupting immune regulatory networks and finally initiating cytokine storms (4).

Pyroptosis, also known as inflammatory necrosis of cells, is a newly discovered programmed cell death different from apoptosis (5). In the canonical pyroptosis pathway, endogenous and exogenous stimuli activate the NOD-like receptor family pyrin domain-containing 3 (NLRP3) inflammasome, which controls the cleavage and activation of CASPASE1 (6). The activated CASPASE1 further drives the cleavage of gasdermin D (GSDMD), releasing its N-terminal fragment, which forms membrane pores and promotes pyroptosis. Meanwhile, activated CASPASE-1 induces the maturation and secretion of pro-inflammatory cytokines, including IL-1β and IL-18 (7).

Amphiregulin (AREG) is a member of the EGF-like family and exists as an inactive, membrane-anchored precursor protein. Intracellular AREG mainly regulates the cell cycle, proliferation, and cytokinesis via unknown mechanisms (8). Alternatively, metalloproteinase-mediated processing releases AREG from the cell surface into the extracellular matrix or allows it to bind EGFR on the surface of neighboring cells during inflammation, controlling the signaling from the EGFR family of receptors (9, 10). Although type II innate lymphoid cells (ILC2s) serve as the primary source of AREG through IL-33-mediated potent upregulation, epithelial and other immune cell types also express AREG during development and tissue homeostasis (11, 12). For example, AREG is significantly expressed in M1 classically polarized macrophages and exerts a protective effect in LPS-induced acute lung injury (13, 14).

Epidermal growth factor receptor (EGFR), a transmembrane receptor tyrosine kinase, plays a vital role in increasing TLR4 cell surface expression and signal transduction in LPS-induced macrophages (15). Another study demonstrated that the EGFR inhibitor erlotinib protects against LPS-induced endotoxicity because TLR4 needs EGFR for signaling (16). In addition, a recent study reports that EGFR promotes pyroptosis in intestinal ischemia–reperfusion injury (17). However, whether EGFR participates in macrophage pyroptosis through extracellular ligand binding remains unclear.

Therefore, this study aims to specifically elucidate the role of extracellular AREG in LPS-induced macrophages. Overall, our findings indicate that RPLP1 regulated AREG secretion at the translation level, aggravating or triggering pyroptosis through autocrine signaling during sepsis development, and extracellular AREG may exert a protective effect on the body through *in vitro* experiments, animal models, and clinical samples. Thus, its regulation of pyroptosis may emerge as a potential therapeutic target for sepsis.

## 2 Materials and methods

### 2.1 Institutional review board statement

This study was approved by the Ethics Committee of the Third Affiliated Hospital of Southern Medical University, Guangzhou, China (No.2023053), and was performed in accordance with the ethical standards of the responsible committee on human experimentation. Serum samples were obtained from the septic patients and healthy donors in the study.

### 2.2 Cell culture and treatment

RAW264.7 macrophages were obtained from the American Type Culture Collection (ATCC, Rockville, MD, USA). Male C57BL/6 mice (8–12 weeks old) were used in all experiments. WT C57BL/6 mice were purchased from the Laboratory Animal Center, Southern Medical University. RAGE knockout mice were obtained from Kanazawa University (Kanazawa, Japan) (18), whereas TLR4, Trif, and Myd88 knockout mice were provided by Professor T.R. Billiar (University of Pittsburgh) (19). RAW264.7 or bone marrow-derived macrophages (BMDMs) were cultured in the DMEM culture medium containing 10% fetal bovine serum at 37°C in an incubator with 5% CO_2_. These macrophages were seeded in 6-well or 12-well culture plates. RAW264.7 was treated with extracellular AREG for varying durations.

To determine the effects of extracellular AREG on LPS (Sigma-Aldrich, #L2630)-induced pyroptosis, BMDMs were isolated, matured in culture, and assigned to the following experimental groups: (1) Control group: BMDMs were stimulated with EGFP (100 ng/mL) for 30 min; (2) AREG group: BMDMs were stimulated with extracellular AREG (100 ng/mL) for 30 min; (3) EGFP+LPS+ATP (Sigma-aldrich,#A6419) group: BMDMs were stimulated with EGFP (100 ng/mL) for 30 min, followed by LPS (1 μg/mL) for 2 h and ATP (5 mM) for 30 min; and (4) AREG+LPS+ATP group: BMDMs were stimulated with extracellular AREG (100 ng/mL) for 30 min, followed by LPS (1 μg/mL) for 2 h and ATP (5 mM) for 30 min.

Additionally, BMDMs were divided into five groups: (1) Ctrl group: no stimulation, (2) LPS group: LPS (1 μg/mL) stimulation for 12 h, (3) Anti-AREG+LPS group: neutralizing antibody of AREG (300 ng/mL, R&D, #AF989) stimulated BMDMs for 1 h, followed by LPS (1 μg/mL) stimulation for 12 h, (4) LPS+ATP group: stimulated with LPS (1 μg/mL) for 2 h, followed by ATP (5 mM) for 30 min, and (5) Anti-AREG+LPS+ATP group: neutralizing antibody of AREG (300 ng/mL) stimulated BMDMs for 1 h, followed by LPS (1 μg/mL) stimulation for 2 h and ATP (5 mM) stimulation for 30 min.

To determine the effect of extracellular AREG on macrophage pyroptosis, BMDMs were divided into six additional groups: (1) Control group: EGFP (100 ng/mL) stimulated BMDMs for 30 min, (2) AREG group: extracellular AREG (100 ng/mL) stimulated BMDMs for 30 min, (3) LPS group: stimulated with LPS (1 μg/mL) for 2 h, (4) EGFP+ATP: stimulated with EGFP (100 ng/mL) for 2.5 h, followed by ATP (5 mM) for 30 min, (5) AREG+ATP group: extracellular AREG (100 ng/mL) stimulated BMDMs for 2.5 h, followed by ATP (5 mM) stimulation for 30 min, and (6) LPS+ATP: LPS (1 μg/mL) stimulated BMDMs for 2 h, followed by ATP (5 mM) stimulation for 30 min. The EGFR inhibitor (Cetuximab) was obtained from Selleck (#A2000), and the NFκB inhibitor (JSH-23) was obtained from MedChemExpress (#HY-13982).

### 2.3 Western blot

BMDMs were washed with Dulbecco’s phosphate-buffered saline (DPBS) and lysed on ice for 30 min using RIPA buffer (Thermo Scientific, #89901). Total protein was extracted, and its concentration was determined using a bicinchoninic acid (BCA) assay (Thermo Scientific, #23227). Proteins (20 μg per sample) were separated via sodium dodecyl sulfate–polyacrylamide gel electrophoresis (SDS-PAGE) and then transferred onto polyvinylidene fluoride membranes (Millipore,#IEVH85R). After blocking, membranes were incubated overnight at 4°C with primary antibodies against rabbit monoclonal anti-EGFR (ABclonal, #A2069), rabbit monoclonal anti-p-EGFR (ABclonal, #A23381), rabbit polyclonal anti-TLR4 (Immunoway, #YT0744), rabbit monoclonal anti-p-IκB (CST, #2859), IκB (CST, #4812), p-P65 (CST, #3033), P65 (CST, #8242), NLRP3 (CST, #15101), GAPDH (CST, #2118), rabbit monoclonal anti-CASPASE1 (AdipoGen, #AG-20B-0042-C100), and rabbit monoclonal anti-GSDMD (Abcam, #ab209845). After incubation with a secondary antibody anti-mouse IgG (Proteintech, #SA00001) or anti-rabbit IgG (CST,#7074s), the protein bands on the membrane were subsequently visualized with an enhanced ECL chemiluminescent substrate (Biosharp, #BL523A).

### 2.4 Cell immunofluorescence

BMDMs were fixed with 4% paraformaldehyde (Beyotime, #P0099), permeabilized with 0.2% Triton X-100 (Beyotime, #P0096), and blocked with 3% BSA (Beyotime, #ST023) at room temperature. After the reaction, cells were incubated with primary antibodies against EGFR (ABclonal, #A23381), TLR4 (Immunoway, #YT0744), or ASC (CST, #67824). Secondary antibody incubation was performed in the dark for 1 h using Alexa Fluor 488-conjugated anti-mouse IgG (Thermo Scientific, #A11029) or Alexa Fluor 594-conjugated anti-rabbit IgG (Thermo Scientific, #11012). The nucleus was counterstained with 4′,6-diamidino-2-phenylindole (Beyotime, #C1006). Immunofluorescence images were captured using LSM 880 with Airyscan.

### 2.5 qRT-PCR analysis

Total RNA was extracted from RAW264.7 using TRIzol reagent (Thermo Scientific, #15596018CN). RNA quantification using NanoDrop (Thermo Scientific), cDNA was synthesized using a ReverTra Ace qPCR RT Kit (Toyobo, #FSQ-201). The qRT-PCR was performed on a 7500 Real-Time PCR System (Applied Biosystems, USA) employing a SYBR Green PCR reagent kit (Dongsheng Biotech).The specific mouse primer sequences were as follows: *Areg* 5′-GCAGATACATCGAGAACCTGGAG-3′ and5′-CCTTGTCATCCTCGCTGTGAGT-3′; *Nlrp3* 5′-TCACAACTCGCCCAAGGAGGAA-3′and5′-AAGAGACCACGGCAGAAGCTAG-3′; *Caspase1* 5′-CTGGGACCCTCAAGTTTTGC-3′ and 5′-GGCAGGCAGCAAATTCTTTC-3′; *Il-1b* 5′-CCCAAGCAATACCCAAAGAA-3′ and 5′-GCTTGTGCTCTGCTTGTGAG-3′. The specific human primer sequences were as follows: *Areg* 5′-GCACCTGGAAGCAGTAACATGC-3′ and 5′-GGCAGCTATGGCTGCTAATGCA-3′; *Egfr* 5′-AACACCCTGGTCTGGAAGTACG-3′ and 5′-TCGTTGGACAGCCTTCAAGACC-3′; *Il-1b* 5′-CCACAGACCTTCCAGGAGAATG-3′ and 5′-GTGCAGTTCAGTGATCGTACAGG-3′; *Il-18* 5′-GATAGCCAGCCTAGAGGTATGG-3′ and 5′-CCTTGATGTTATCAGGAGGATTCA-3′. 18S 5′-AGTCCCTGCCCTTTGTACACA-3′ and 5′-CGATCCGAGGGCCTCACTA-3′. The expression of these genes was normalized with 18S ribosomal RNA via the relative CT value.

### 2.6 Isolation of RNC mRNA

Cycloheximide (Sigma, #C7698) was added to the culture medium at a final concentration of 100 μg/mL 15 min before cell collection. Cells were lysed on ice using a ribosome-specific lysis buffer. The lysate was centrifuged at 13,200 rpm for 10 min at 4°C to remove cellular debris, and the supernatant was transferred to prechilled ultracentrifuge tubes (Beckman, #344059) balanced with 30% sucrose solution. Ultracentrifugation was performed at 185,000 ×g for 5 h at 4°C to pellet ribosome–nascent chain complexes (RNCs). After discarding the sucrose solution, the RNC pellet was resuspended in ribosome buffer and sonicated on ice to ensure total dissolution of complexes. RNC-derived mRNA was extracted using TRIzol reagent and prepared for subsequent qRT-PCR analysis.

### 2.7 ELISA

AREG secretion in the cellular supernatant, as well as in the serum of mice or patients with sepsis, was quantified using an ELISA kit from R&D Systems (#989-AR). TNFα (#EMC102), IL-6 (#EMC004), CCL2 (#EMC113), and CCL3 (#EMC010) in the cellular supernatant were quantified using ELISA kits from QuantiCyto. IL-1β (#E-EL-M0037) and IL-18 (#E-EL-M0730) were quantified with ELISA kits from Elabscience. These levels of cytokines were measured according to the protocols of the manufacturer. The concentration of cytokines was calculated based on the standard curve.

### 2.8 Purification of extracellular AREG

In brief, the extracellular segment of AREG, excluding the signal peptide, transmembrane, and intracellular regions, was cloned into a His-tagged pET14b vector using the subcloning method. The recombinant pET14b-AREG was transformed into competent *E. coli* BL21 cells to obtain extracellular AREG protein, which was purified with Ni-NTA Sepharose chromatography (Macherey-Nagel, #2403-001). EGFP protein was purified as a control for AREG. The obtained recombinant protein AREG was then identified using SDS-PAGE, and the endotoxin removal was performed using endotoxin removal gel (Thermo Scientific, #20340). An endotoxin assay kit (Thermo Scientific, #Q32891) was used to detect endotoxin levels in recombinant AREG and EGFP proteins.

### 2.9 Coimmunoprecipitation

RAW264.7 cells were treated with His-AREG (100 ng/mL) for 3 h. Then, cells were lysed and collected with lysis buffer (Thermo Scientific, #87787), and protein concentration was quantified with a BCA kit. Total IP protein (1 mg) was incubated overnight at 4°C with A/G magnetic beads (Thermo Scientific, #80003) and either an anti-His Tag Monoclonal Antibody (Thermo Scientific, #MA1-21315) or a Mouse IgG Isotype Control (MCE, #HY-P80757). After washing the magnetic beads with DPBS, the samples were dissolved in SDS-PAGE loading buffer and subjected to Western blot analysis with the anti-His, TLR4 (ABclonal, #A11226), or EGFR (ABclonal, #A2069) antibodies.

### 2.10 Electron microscopy image

The BMDMs were collected into a centrifuge tube, and after discarding the supernatant, they were fixed in 2.5% glutaraldehyde while avoiding resuspension and agitation. The samples were incubated at room temperature for 1 h, after which glutaraldehyde was discarded, and DPBS was added. Electron microscopy imaging was conducted at the Central Laboratory of Southern Medical University.

### 2.11 Isolation of monocytes in patients with sepsis

Monocytes were isolated from healthy individuals, as well as patients with general and severe sepsis, using a specific isolation kit (TBD Science, #TBD2011H). Criteria for the classification of patients with sepsis were based on a previously published study (20).

### 2.12 Statistical analysis

Data were presented as mean ± standard deviation. Statistical analyses were performed using GraphPad Prism (GraphPad Software, version 9), with experiments repeated at least three times. An unpaired two-tailed t-test was used for comparisons between the two groups. One-way analysis of variance followed by the Bonferroni *post-hoc* test was used for multiple group comparisons. Statistical significance was defined as p < 0.05. Restricted cubic splines (RCS) and segmented linear regression analyses were performed using R software (version 4.2.2) and MSTATA software (www.mstata.com).

## 3 Results

### 3.1 Dynamic expression of AREG in sepsis

**Figure 1:**
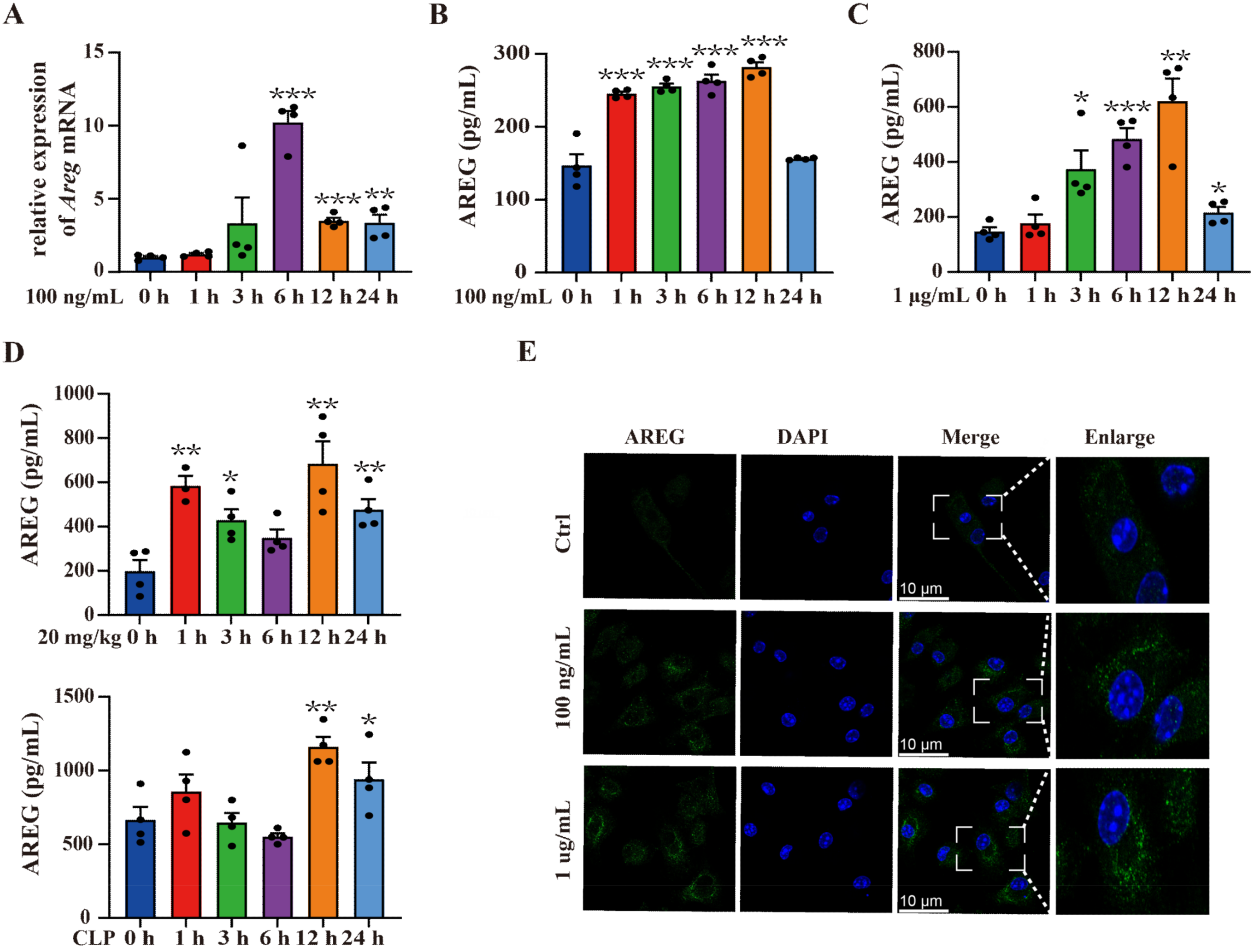
Dynamic expression of AREG in sepsis. RAW264.7 cells were stimulated with LPS (100 ng/mL or 1 μg/mL) for 1, 3, 6, 12, and 24 h. AREG mRNA and protein expressions were detected using RT-PCR and ELISA **(A-C)**. WT C57BL/6 mice were intraperitoneally injected with LPS (20 mg/kg) and constructed with the CLP model, whereas AREG protein expression in serum was detected via ELISA **(D)**. BMDM was stimulated with LPS (100 ng/mL or 1 μg/mL) for 12 h, and AREG protein expression was detected via Immunofluorescence **(E)**. Data presented as mean ± SEM (n ≥ 3). *P < 0.05, **P < 0.01, ***P < 0.001 vs. Control. AREG, amphiregulin; WT, wild type; BMDM, bone marrow-derived macrophages; CLP, cecal ligation and puncture.

To evaluate the AREG expression in LPS-induced RAW264.7 macrophages, we detected AREG mRNA expression at 1, 3, 6, 12, and 24 h after LPS stimulation. AREG expression rapidly peaked at 6 h and subsequently declined (**Figure 1A**). To verify these findings, we compared AREG protein expression in the culture supernatant of RAW264.7. The AREG gene and protein levels almost peaked at 6 h post-LPS stimulation (**Figure 1B**). In addition, exposure to higher concentrations of LPS resulted in greater AREG protein release (**Figure 1C**). The significantly high expression of AREG protein was also detected in the serum of septic mice and BMDMs (**Figures 1D, E**).

### 3.2 RPLP1 regulates LPS-induced AREG expression at the translational level

**Figure 2:**
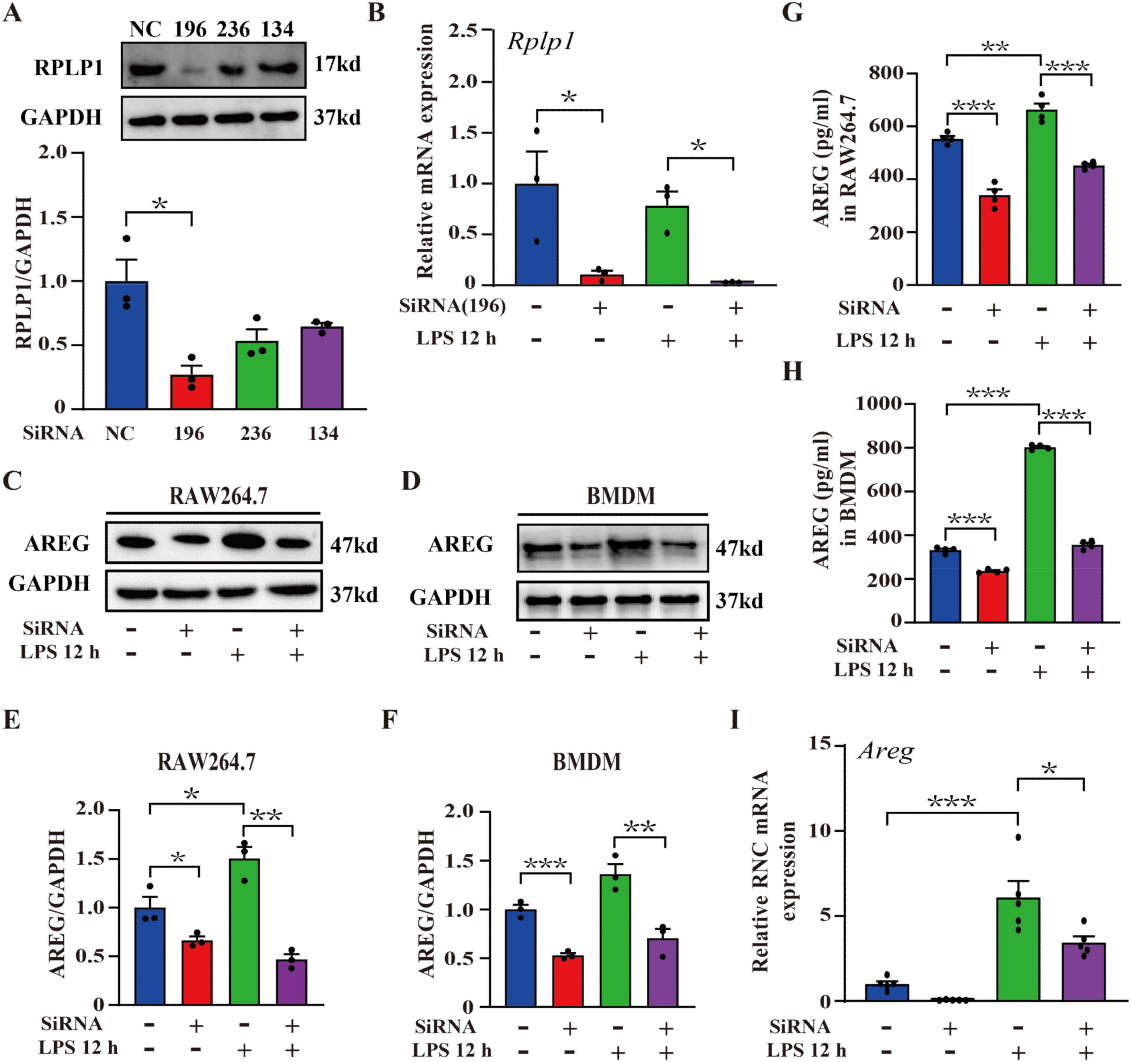
RPLP1 regulates LPS-induced AREG expression at the translational level. RAW264.7 macrophages were transfected with three different siRNAs (196, 236, and 134) targeting *Rplp1*, and the protein expression of RPLP1 was detected via Western blot **(A)**. *RPLP1* mRNA expression was detected via RT-PCR following LPS stimulation (100 ng/mL) for 12 h **(B)**. AREG expression in whole-cell lysates **(C-F)** and culture supernatant **(G, H)** of LPS-stimulated RAW264.7 and BMDM was detected after RPLP1 knockdown with interference probe 196 assessed by Western blot and ELISA. AREG expression in the ribosome nascent chain complex of LPS-stimulated RAW264.7 was detected after RPLP1 was knocked down with interference probe 196 via RT-PCR **(I)**. Data are presented as mean ± SEM (n ≥ 3). *P < 0.05, **P < 0.01, ***P < 0.001 vs. Control. AREG, amphiregulin; BMDM, bone marrow-derived macrophages.

AREG, a secreted transmembrane protein, is synthesized by endoplasmic reticulum-bound ribosomes and then transported to its final destination via the secretory pathway. During translation, its N-terminal signal peptide, emerging from the ribosome, is recognized (21). A study reports that AREG is a translational target of RNA helicase DDX3, showing the presence of 29 ribosomal proteins interacting with DDX3 (22). Therefore, we hypothesized that specific ribosomal proteins may regulate AREG translation in LPS-induced macrophages. Under inflammatory cytokine stimulation (e.g., IFNγ and TNFα), ribosomal protein RPLP1 selectively enhances the translation of mRNAs encoding secretory or transmembrane proteins, including HLA, TAP1, and TAP2 (23). To investigate whether RPLP1 regulates AREG translation, we first performed a knockdown assay in RAW264.7 macrophages. We designed three siRNA sequences targeting RPLP1. The siRPLP1-196 significantly suppressed RPLP1 expression at the protein and mRNA levels in LPS-induced RAW264.7 macrophages. Thus, siRPLP1-196 was selected for subsequent experiments (**Figures 2A, B**). We further knocked down RPLP1 by applying the 196 probes to detect the expression of AREG in LPS-induced macrophages. RPLLP1 knockdown significantly reduced AREG expression in intracellular and culture supernatant fractions of LPS-stimulated RAW264.7 macrophages and BMDMs (**Figures 2C–H**). To analyze translation-level changes in AREG expression following RPLP1 knockdown, RNC-mRNA was separated from other cellular components using sucrose density gradient centrifugation. Knocking down RPLP1 significantly inhibited the LPS-induced AREG translation (**Figure 2I**). Collectively, these findings indicate that ribosomal protein RPLP1 mediates the translational regulation of AREG expression in LPS-induced macrophages.

### 3.3 Extracellular AREG induces NFκB activation through the EGFR/TLR4 signaling pathway in macrophages

**Figure 3:**
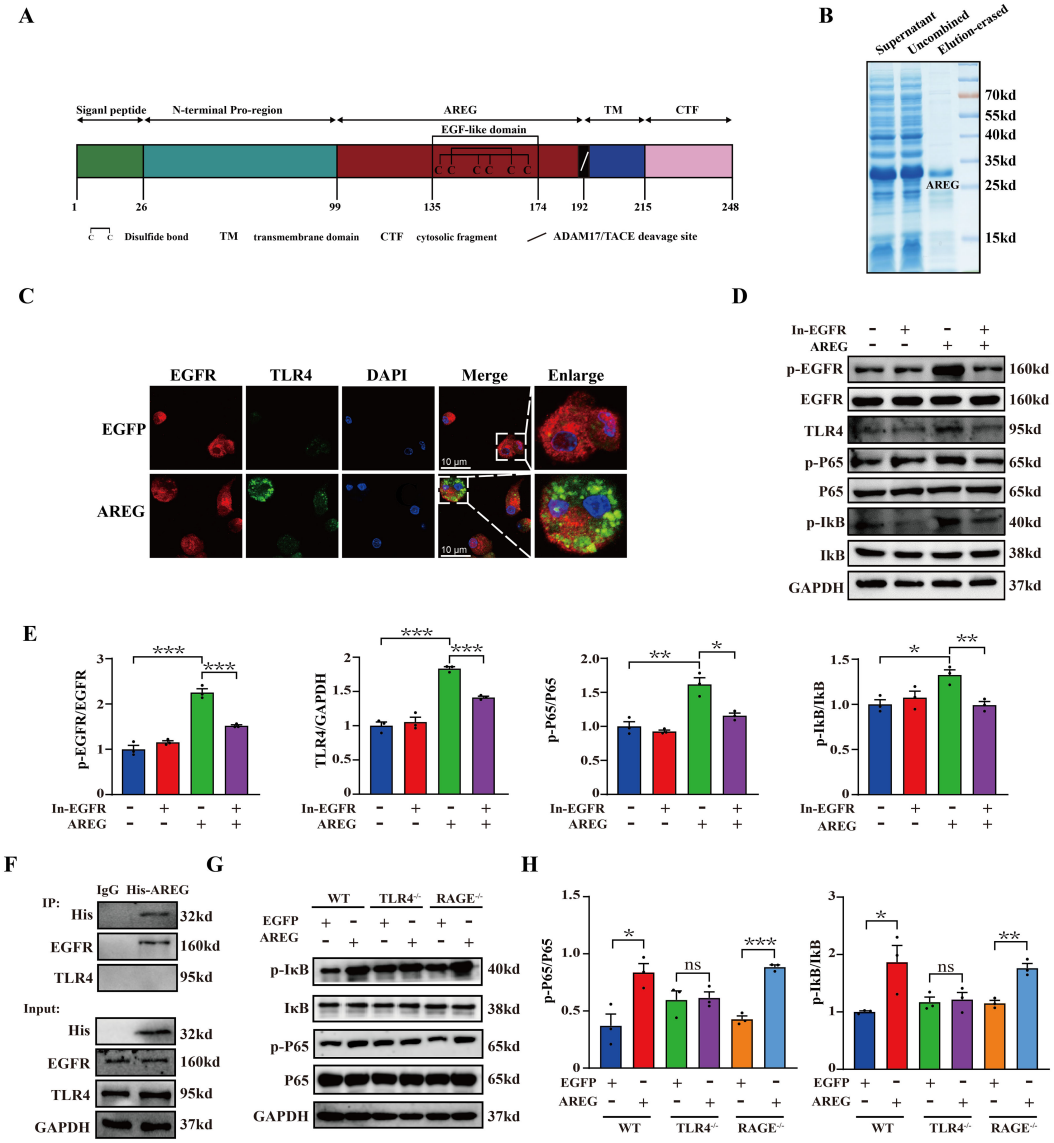
EGFR inhibition and TLR4 silencing impair extracellular AREG-induced IκB phosphorylation and NFκB activation in BMDMs. Structural features of the AREG protein were analyzed using DOG2.0 software **(A)**. Purification of extracellular AREG was performed via Coomassie Blue staining **(B)**. EGFR and TLR4 expression was detected in extracellular AREG-stimulated BMDM via Immunofluorescence **(C)**. p-EGFR, TLR4, p-P65, and p-IκB expression levels were detected in inhibitor of EGFR (1 mM) pretreating BMDM for 4 h through Western blot **(D, E)**. RAW264.7 were collected for lysis after treatment with His-AREG for 3 h. Immunoprecipitation was performed with a specific antibody against the His tag, EGFR, and TLR4 to assess the interaction between extracellular AREG and EGFR **(F)**. p-P65 and p-Iκb expression levels were detected in TLR4^−/−^BMDM and RAGE^−/−^BMDM using Western blot **(G, H)**. Data are presented as mean ± SEM (n ≥ 3). *P < 0.05, **P < 0.01, ***P < 0.001 vs. Control. AREG, amphiregulin; BMDM, bone marrow-derived macrophages; EGFR, epidermal growth factor receptor.

EGF family members, including EGF and TGFα, are known to activate NFκB (24, 25). Whether AREG also contributes to NFκB activation remains unclear. To elucidate the critical role of AREG in LPS-induced macrophages, we subcloned a soluble extracellular AREG without the signal peptide, transmembrane, and intracellular segments (**Figures 3A, B**). **Figures 3C–E** shows that extracellular AREG induced significant expressions of TLR4 and phosphorylated EGFR, IκB, and P65 in BMDMs. These effects were attenuated EGFR kinase inhibition. Consistent with previous findings, the TLR4 knockout inhibited the extracellular AREG-induced phosphorylation of IκB and P65 (**Figures 3G, H**). We further confirmed by coimmunoprecipitation that the extracellular AREG specifically binds to EGFR but not to TLR4 in macrophages (**Figure 3F**). Another potential mechanism for extracellular AREG-induced NFκB activation involves an alternative membrane receptor. Ligand-activated RAGE increases inflammation by binding and activating EGFR (26). However, extracellular AREG-induced phosphorylation of IκB and P65 remained unaffected by RAGE knockout (**Figures 3G, H**). These findings suggest that EGFR/TLR4 is vital for extracellular AREG-induced NFκB activation in BMDMs.

### 3.4 Extracellular AREG aggravated inflammatory response and LPS-induced pyroptosis in macrophages

**Figure 4:**
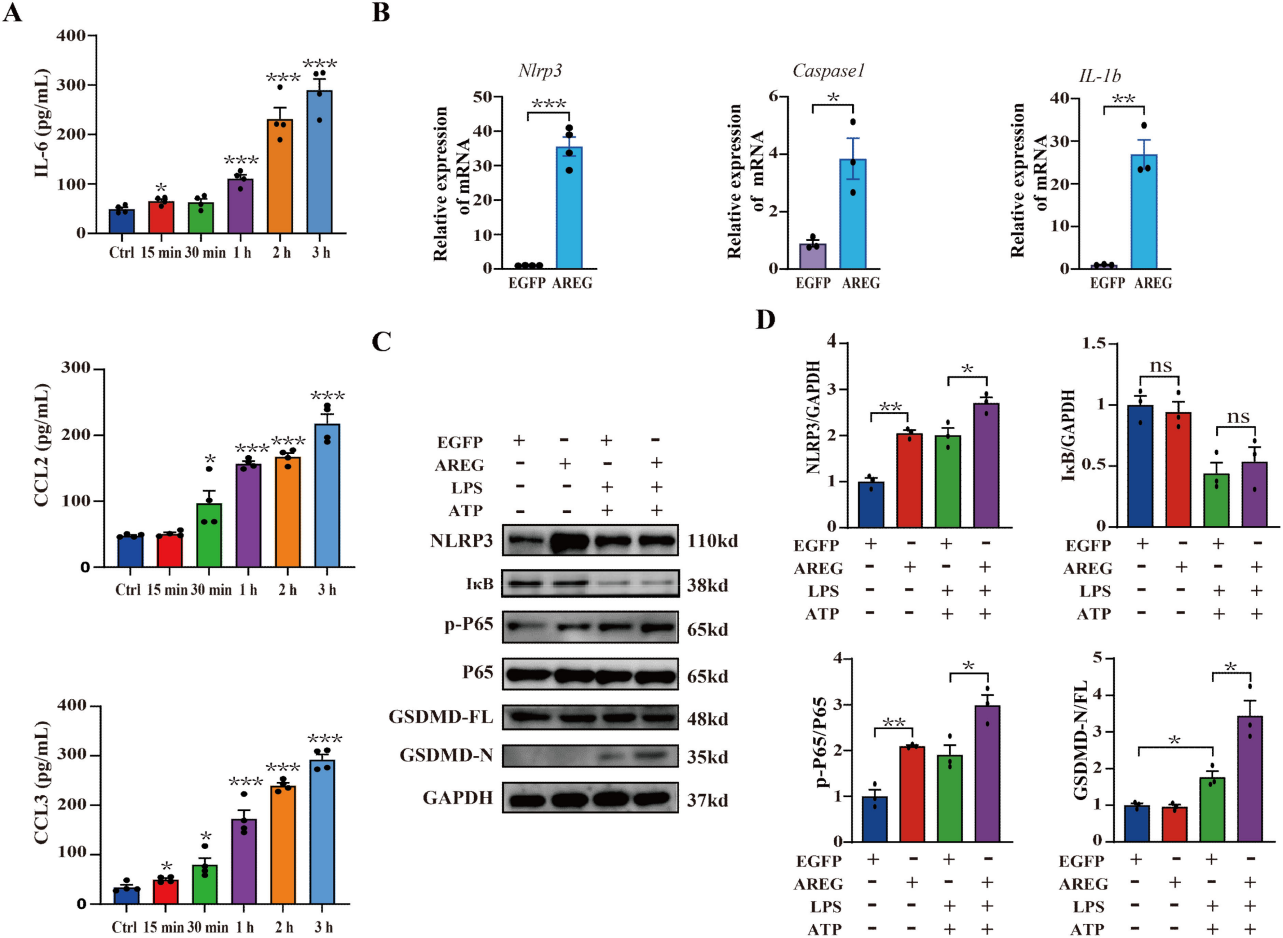
Extracellular AREG aggravates the inflammatory response and LPS-induced pyroptosis in macrophages. RAW264.7 cells were stimulated with AREG (100 ng/mL) for 15 min, 30 min, 1 h, 2 h, and 3 h, and the expression of supernatant IL-6, CCL2, and CCL3 was detected via ELISA **(A)**. RAW264.7 cells were stimulated with AREG (100 ng/mL) for 1 h, and the expression of *Nlrp3, Caspase1, and IL1β* mRNA was detected via RT-PCR **(B)**. LPS-stimulated BMDM was pretreated with AREG, and expression of NLRP3, p-P65, and GSDMD-N was detected via Western blot **(C, D)**. ns, no significant; AREG, amphiregulin; GSDMD, gasdermin D.

The activation of NFκB induces the expression of multiple genes and promotes the production of multiple cytokines involved in the inflammatory response (27). We first observed that extracellular AREG rapidly induced the secretion of IL-6, CCL2, and CCL3 in RAW264.7 macrophages (**Figure 4A**) but did not affect TNFα secretion (Not shown). Another EGF family member, TGFα, inhibits microglial pyroptosis in demyelinating diseases through the NFκB pathway (27, 28). Therefore, determining whether extracellular AREG is involved in pyroptosis is essential. Extracellular AREG stimulation significantly upregulated the expression of *Nlrp3, Caspase1, and IL-1β* mRNA in RAW264.7 macrophages (**Figure 4B**). Since macrophage pyroptosis is often studied using an LPS and ATP co-stimulation model (29, 30), we examined the effect of extracellular AREG on pyroptosis in LPS+ATP-stimulated BMDMs. Pyroptosis can be mediated by the effector molecule GSDMD, which is cleaved by CASPASE1 (31). **Figures 4C, D** show that LPS and ATP co-stimulation significantly increased the expression of p-P65 and GSDMD-N while promoting IκB degradation. Extracellular AREG pretreatment further upregulated the expression of p-P65 and GSDMD-N but did not affect IκB degradation. These findings confirm that extracellular AREG directly induces an inflammatory response and may accelerate macrophage pyroptosis via NFκB activation.

### 3.5 EGFR inhibiting and TLR4 silencing inhibit extracellular AREG-induced macrophages pyroptosis

**Figure 5:**
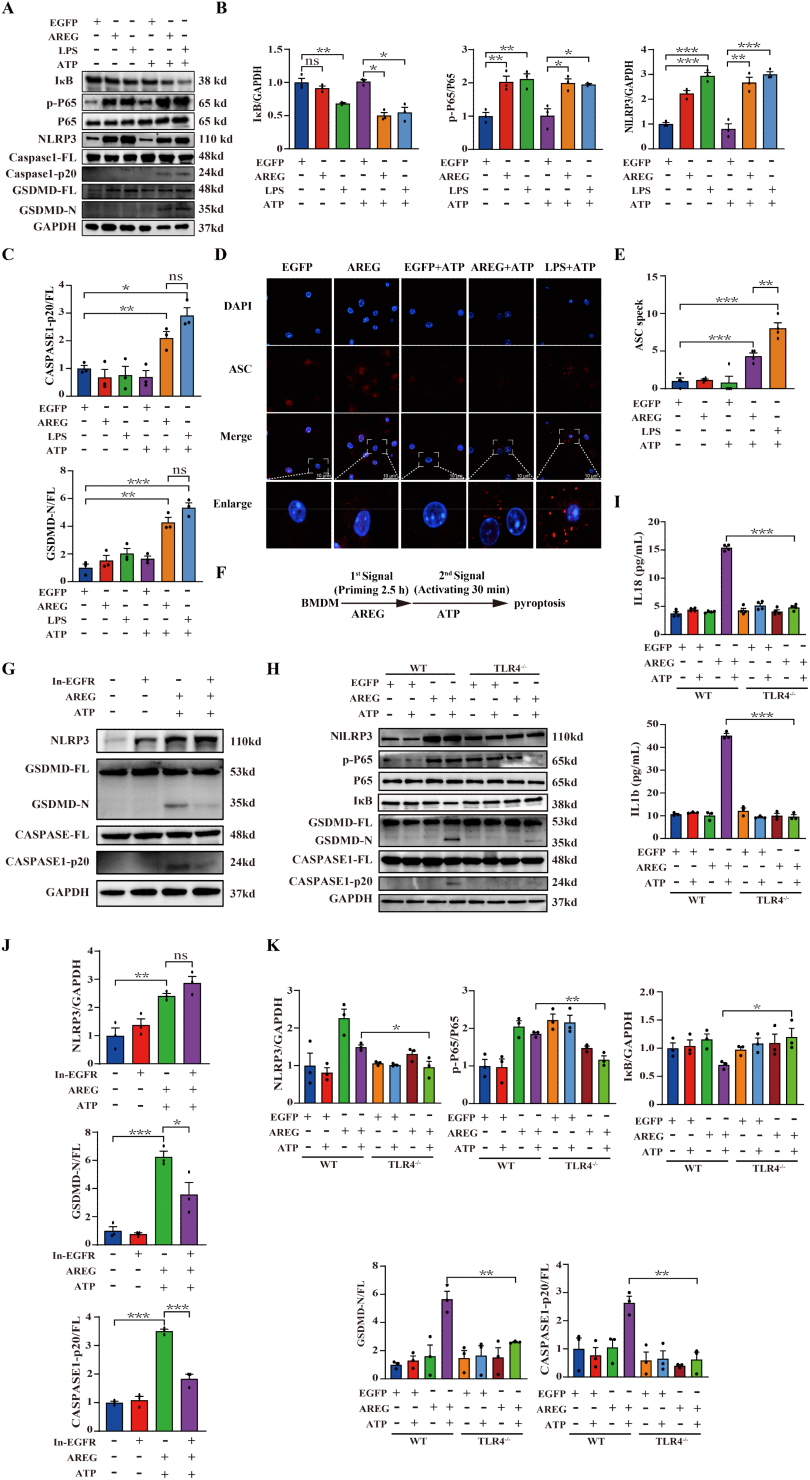
EGFR inhibition and TLR4 silencing impair AREG-induced macrophage pyroptosis. BMDM was stimulated with AREG+ATP or LPS+ATP, and the expression of NLRP3, p-P65, p-IκB, CASPASE-1-p20, and GSDMD-N was detected via Western blot **(A-C)**. BMDM was stimulated with AREG+ATP or LPS+ATP, and oligomerization of ASC was detected using immunofluorescence **(D, E)**. Experimental diagram of AREG-induced macrophage pyroptosis. For the priming step, BMDM was treated with AREG for 2.5 as the first signal and the ATP as the second signal **(F)**. NLRP3, CASPASE-1-p20, and GSDMD-N expressions were detected in the EGFR inhibitor (1 mM) pretreating AREG +ATP-induced BMDM for 4 h through Western blot **(G, J)**. NLRP3, CASPASE-1-p20, and GSDMD-N expressions were detected in AREG +ATP-induced TLR4^−/−^BMDM via Western blot **(H, K)**. The expression of IL-1b and IL-18 was detected in the supernatant of AREG +ATP-induced TLR4^−/−^BMDM via ELISA **(I)**. Data are presented as mean ± SEM (n ≥ 3). *P < 0.05, **P < 0.01, ***P < 0.001 vs. Control. ns, no significant; AREG, amphiregulin; BMDM, bone marrow-derived macrophages; EGFR, epidermal growth factor receptor; GSDMD, gasdermin D; ATP, adenosine triphosphate.

Based on the above findings, we hypothesized that extracellular AREG combined with ATP induces macrophage pyroptosis via the EGFR/TLR4 signaling module. To confirm this hypothesis, we stimulated BMDMs with extracellular AREG and ATP. Cotreatment with extracellular AREG and ATP upregulated p-P65 expression and promoted IκB degradation BMDMs. Furthermore, extracellular AREG and ATP cotreatment significantly promoted the expression of NLRP3, GSDMD-N, and CASPASE1-p20. However, extracellular AREG alone did not increase the expression of CASPASE1-p20 or GSDMD-N (**Figures 5A–C**). The adaptor protein ASC facilitates caspse-1 activation by forming a multi-protein complex to activate with its precursor (31, 32). Consistent with these findings, extracellular AREG +ATP and LPS +ATP also induced ASC oligomerization in BMDMs (**Figures 5D, E**). This suggests that extracellular AREG can induce macrophage pyroptosis by activating NFκB.

To investigate the role of the EGFR/TLR4/Myd88/NFκB pathway in the extracellular AREG-induced pyroptosis (**Figure 5F**), we first pretreated extracellular AREG-induced pyroptosis with the EGFR kinase inhibitor. **Figures 5G, J** show that the EGFR kinase inhibition decreased the expression of GSDMD-N and CASPASE1-p20 in extracellular AREG-induced pyroptosis. However, no significant difference was observed in the expression of NLRP3 after EGFR kinase inhibition. TLR4 depletion diminished extracellular AREG-induced expression p-P65, NLRP3, GSDMD-N, and CASPASE-1-p20, as well as IκB degradation in BMDMs (**Figures 5H, K**). Additionally, TLR4 depletion in extracellular AREG-induced pyroptosis significantly reduced the secretion of IL-1β and IL-18 (**Figure 5I**).

### 3.6 Neutralizing extracellular AREG decreases LPS-induced TLR4 expression and pyroptosis in macrophages

**Figure 6:**
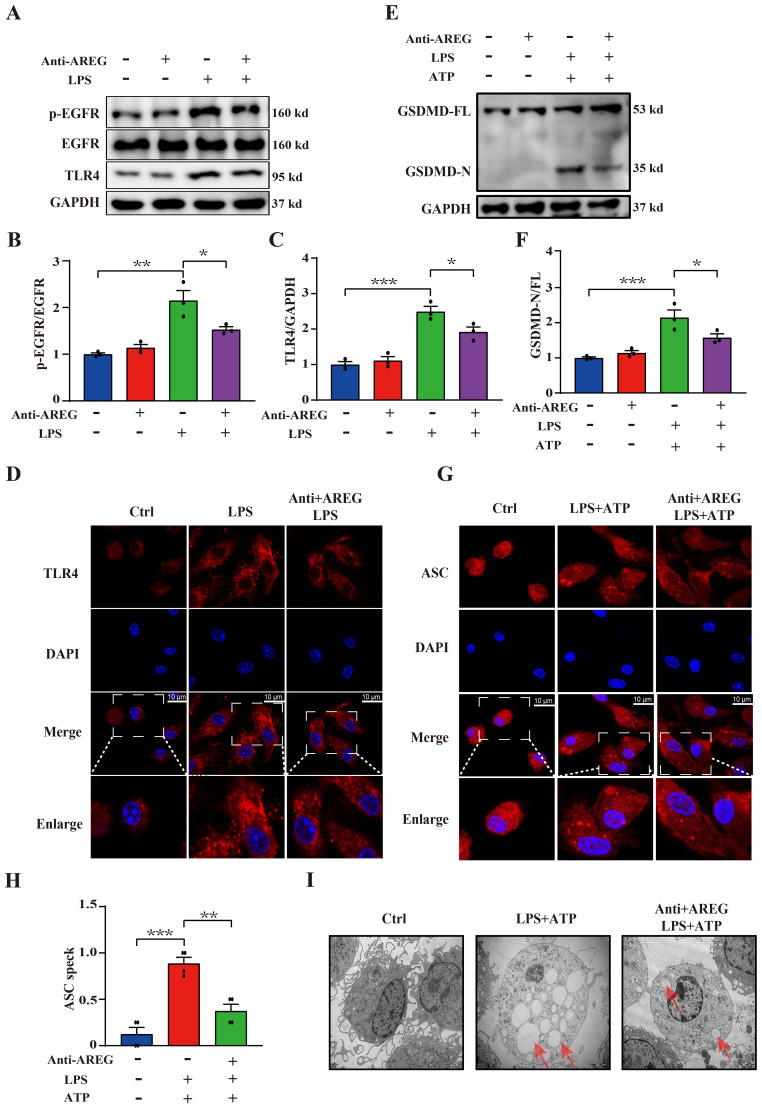
Neutralizing extracellular AREG decreases LPS-induced TLR4 expression and pyroptosis in macrophages. LPS-induced BMDM was pretreated with a neutralizing antibody of AREG. p-EGFR, TLR4, and GSDMD-N expression levels were detected via Western blot and immunofluorescence **(A-C, E, F)**, TLR4 expression and ASC oligomerization was detected through immunofluorescence **(D, G, H)**. Formation of pyrosomes (red arrows) was detected using electron microscopy, scale bars, 2 μm **(I)**. Data are presented as mean ± SEM (n ≥ 3).*P < 0.05, **P < 0.01, ***P < 0.001 vs. Control. AREG, amphiregulin.

Next, we examined whether extracellular AREG contributes to LPS-mediated activation of EGFR tyrosine kinase activity and TLR4 expression. We found that AREG neutralization reduced the expression of p-EGFR and TLR4 (**Figures 6A–D**), suggesting that AREG depletion contributes, at least in part, to the reduction in EGFR tyrosine kinase activity and TLR4 expression in LPS-induced macrophages. Therefore, we investigated whether extracellular AREG influences LPS-induced macrophage pyroptosis. AREG neutralization significantly reduced the expression of GSDMD-N (**Figures 6E, F**), oligomerization of ASC (**Figures 6G, H**), and formation of pyroptosome in LPS-induced macrophage pyroptosis (**Figure 6I**). These findings indicate that extracellular AREG links the TLR4 pathway to EGFR activation in macrophage pyroptosis.

### 3.7 MyD88 silencing and NFκB inhibiting restrain extracellular AREG-induced macrophage pyroptosis

**Figure 7:**
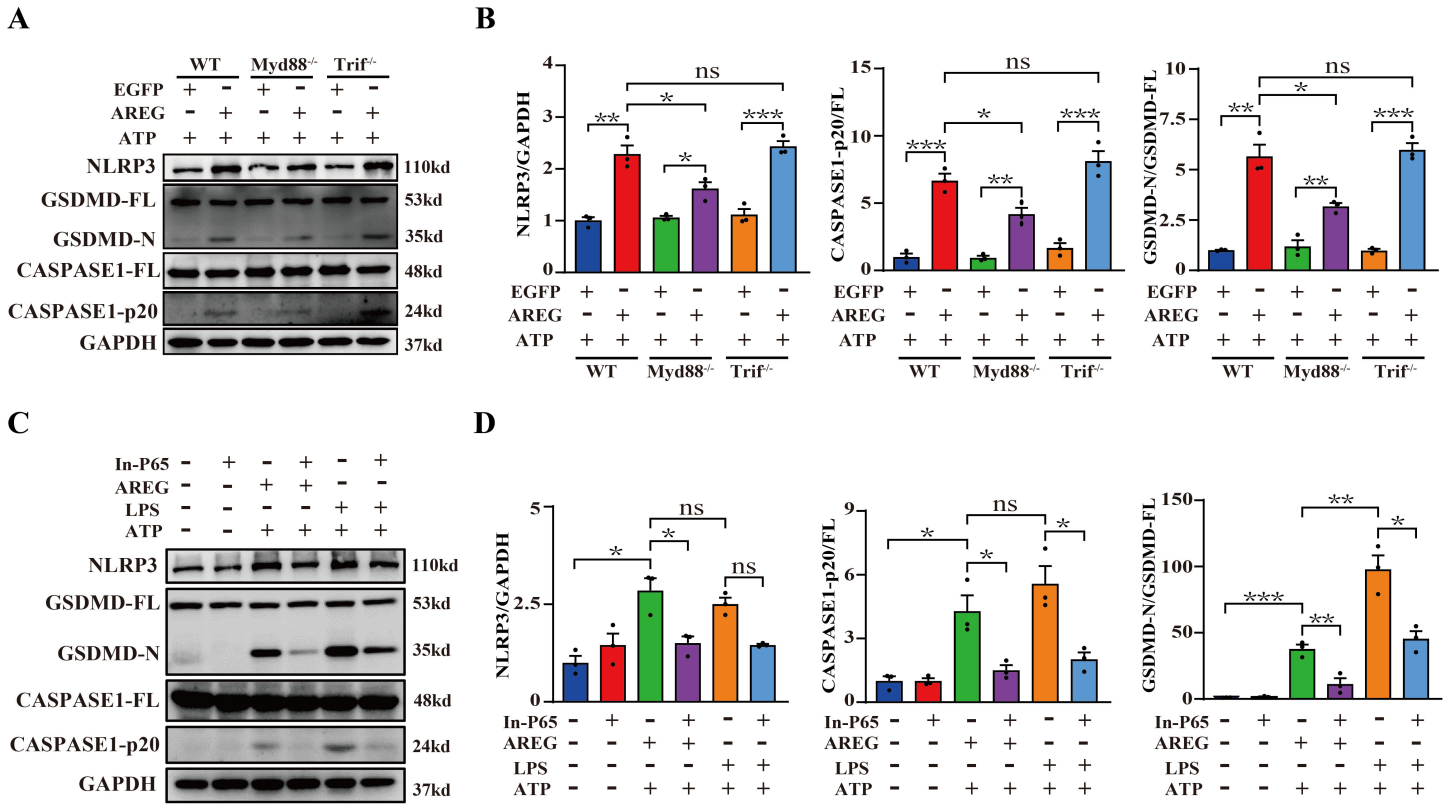
MyD88 silencing and NFκB inhibition restrain extracellular AREG-induced macrophage pyroptosis. NLRP3, CASPASE1-p20, and GSDMD-N expression levels were detected in extracellular AREG-induced Myd88^−/−^ and Trif^−/−^BMDM via Western blot **(A, B)**. NLRP3, CASPASE1-p20, and GSDMD-N expression levels were detected in the inhibitor of NFκB (P65) (20 μM) pretreating extracellular AREG-induced BMDM for 2 h via Western blot **(C, D)**. Data are presented as mean ± SEM (n ≥ 3). *P < 0.05, **P < 0.01, ***P < 0.001 vs. Control. ns, no significient; AREG, amphiregulin; BMDM, bone marrow-derived macrophages; EGFR, epidermal growth factor receptor; GSDMD, gasdermin D.

TLR4 signaling activates the translocation of NFκB into the nucleus through MyD88-dependent and TRIF-dependent pathways (33). We further investigated the effects of TLR4 downstream signaling in extracellular AREG-induced macrophage pyroptosis, and the results showed that MyD88 knockout, but not TRIF, significantly decreased the expression of NLRP3, GSDMD-N, and CASPASE-1-p20 in extracellular AREG-induced BMDM pyroptosis (**Figures 7A, B**). Additionally, NFκB inhibition downregulated the expression of NLRP3, GSDMD-N, and CASPASE-1-p20 in extracellular AREG-induced and LPS-induced BMDM pyroptosis (**Figures 7C, D**).

### 3.8 DTT-pretreated extracellular AREG restrains macrophage pyroptosis

**Figure 8:**
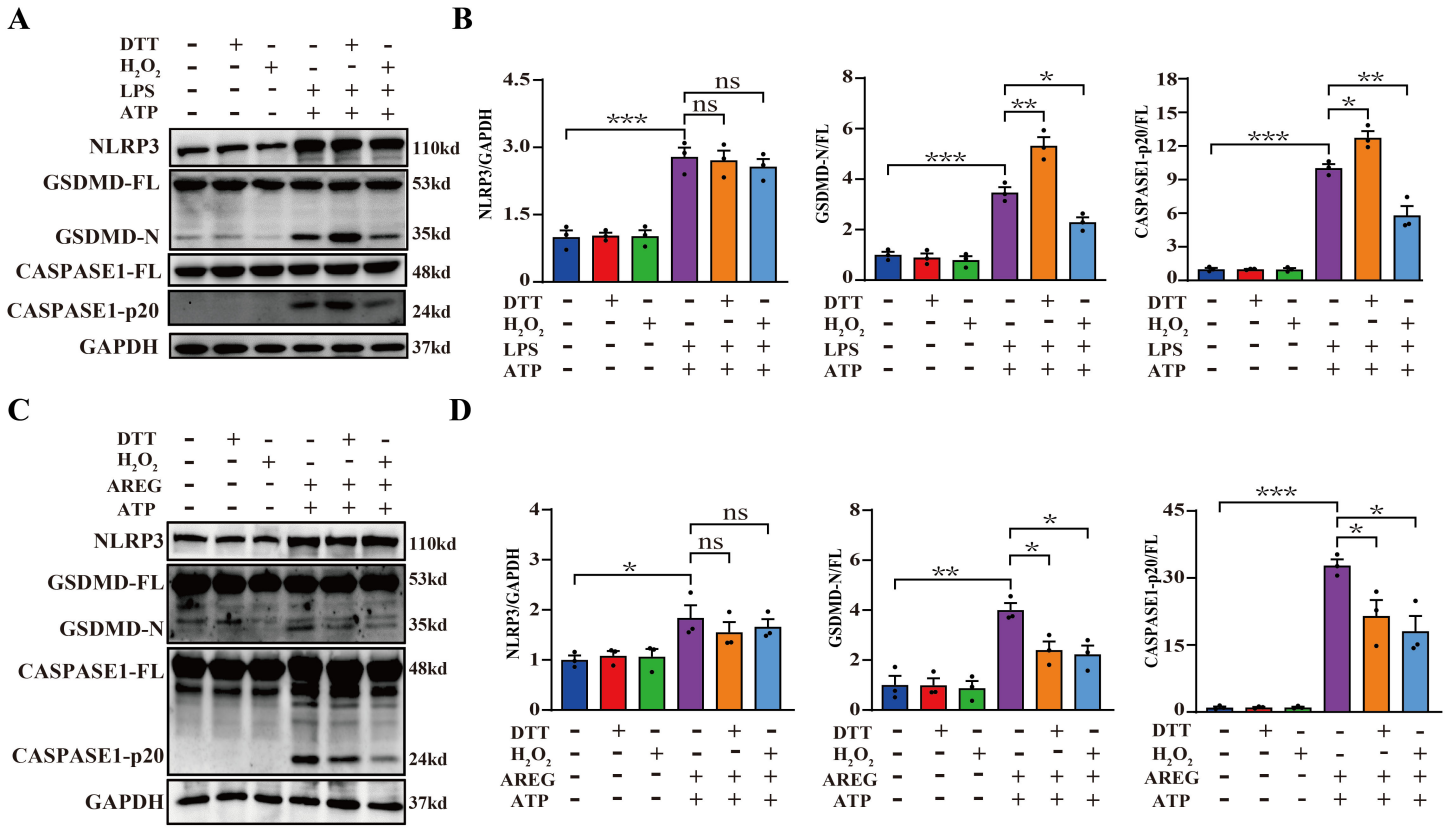
DTT-pretreated extracellular AREG restrains macrophage pyroptosis. NLRP3, CASPASE1-p20, and GSDMD-N expressions were detected in LPS-induced BMDM after DTT (1 mM) or H_2_O_2_ (100 μM) pretreating LPS for 1 h **(A, B)**. NLRP3, Caspase1-p20, and GSDMD-N expression were detected in AREG-induced BMDM after DTT (1 mM) or H_2_O_2_ (100 μM) pretreating AREG for 1 h **(C, D)**. Data are presented as mean ± SEM (n ≥ 3).*P < 0.05, **P < 0.01, ***P < 0.001 vs. Control. ns, no significient; AREG, amphiregulin; BMDM, bone marrow-derived macrophages; EGFR, epidermal growth factor receptor; GSDMD, gasdermin D.

The inner ring structure of the disulfide bond within the EGF family member domain serves as the receptor-binding region necessary for biological activity. Extracellular AREG is initially synthesized as a membrane-bound precursor, which undergoes proteolytic cleavage to release a soluble EGF domain containing a disulfide bond, enabling its extracellular function (33, 34). Therefore, we pretreated extracellular AREG or LPS with either a reducing agent (DTT) or oxidizing agent (H_2_O_2_) before stimulating BMDMs, followed by ATP reduction. LPS pretreated with DTT significantly increased expression of GSDMD-N and CASPASE1-p20, whereas LPS pretreated with H_2_O_2_ significantly inhibited macrophage pyroptosis (**Figures 8A, B**). Furthermore, extracellular AREG pretreated with DTT or H_2_O_2_ downregulated the expression of GSDMD-N and CASPASE1-p20 in macrophage pyroptosis (**Figures 8C, D**). In addition, neither DTT nor H_2_O_2_ treatment affected the expression of NLRP3 in AREG-treated or LPS-treated macrophages. These findings suggest that DTT may specifically reduce the disulfide bond in the extracellular domain of AREG, thereby inhibiting extracellular AREG-induced macrophage pyroptosis.

### 3.9 Extracellular AREG mediates sepsis in mice and humans

**Figure 9:**
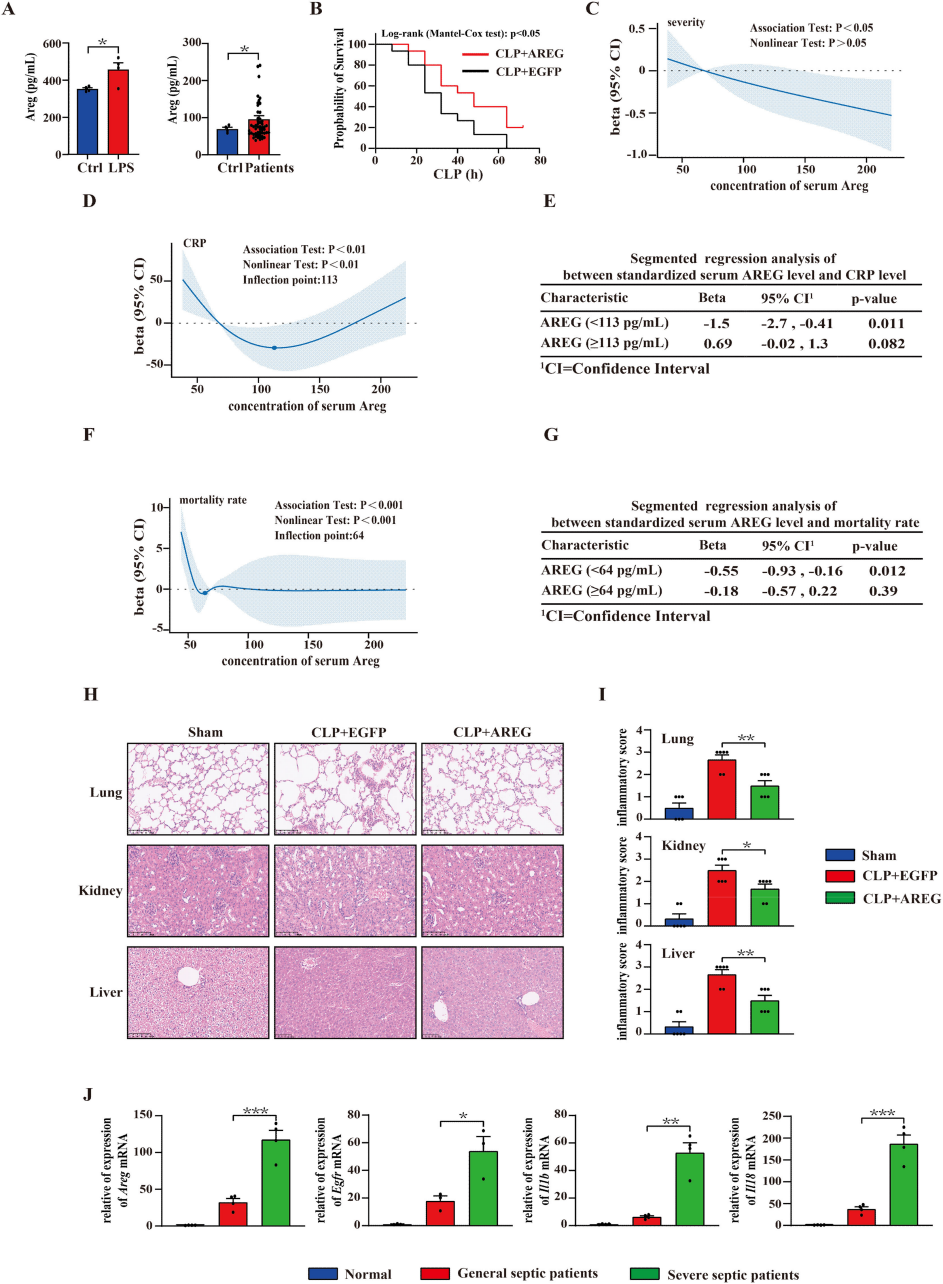
Serum AREG levels correlate with sepsis severity and mortality. Thp1 was stimulated with LPS (100 ng/mL) for 6 h, and supernatant AREG expression was detected via ELISA. Serum AREG in patients with sepsis was detected using ELISA **(A)**. Administration of extracellular AREG (5 μg per mouse, tail vein injection) 30 min before severe CLP-induced septic mice, n = 15 mice/group **(B)**. The association of concentration of serum AREG among CRP level, severity, and mortality rates in patients with sepsis was represented with the RCS function, where the beta on the Y-axis represents the relative strength and direction of influence of independent variable on a dependent variable **(C, D, F)**. Segmented linear association analysis was performed between the standardized serum AREG and CRP levels, as well as between the standardized serum AREG and mortality rates in patients with sepsis **(E, G)**. Representative H&E staining of lung, kidney, and liver at 12 h after extracellular AREG pretreated-CLP septic mice. The inflammatory cell infiltrate score reflects tissue injury in CLP mice **(H, I)**. RT-PCR was used to detect the expression of genes of the Areg-mediated pyroptosis signaling pathway in patients with sepsis **(J)**. Data are presented as mean ± SEM (n ≥ 3). *P < 0.05, **P < 0.01, ***P < 0.001. AREG, amphiregulin; CLP, cecal ligation and puncture; RCS, restricted cubic splines.

To evaluate the significance of serum AREG in patients with sepsis, we first examined its expression in LPS-stimulated THP1 and in patients with sepsis. AREG was highly expressed in the culture supernatant of LPS-stimulated THP1 cells and the serum of patients with sepsis (**Figure 9A**). The cecal ligation and puncture (CLP) model is the most widely utilized and well-established sepsis model, closely resembling human sepsis and suitable for investigating potential mechanisms and therapeutic strategies. A study shows that AREG can alleviate LPS-induced acute lung injury (12). Based on this, we explored the effect of extracellular AREG on sepsis progression. Pretreatment with extracellular AREG lowered CLP-induced mortality (**Figure 9B**). Besides, we conducted a clinical study involving 54 patients diagnosed with general sepsis, severe sepsis, or septic shock. Using RCS, we analyzed the association between serum AREG levels, serum C-reactive protein (CRP) concentration, disease severity, and sepsis-related mortality.

The analysis of RCS revealed a significant overall correlation between serum AREG levels, sepsis severity, and mortality. The risk of sepsis exacerbation alleviated as AREG concentrations increased (**Figure 9C**). When AREG concentrations were <113 pg/mL, CRP levels decreased with increasing AREG concentrations. However, when AREG concentrations exceeded 113 pg/mL, CRP levels showed no significant change (**Figures 9D, E**). In addition, when AREG concentrations were below 64 pg/mL, the risk of death rapidly decreased with increasing AREG concentrations, but no significant change was observed beyond this threshold (**Figures 9F, G**).

Furthermore, histopathological analysis of the lungs, kidneys, and liver revealed that extracellular AREG administration suppressed inflammatory cell infiltration (**Figures 9H, I**). We further explored whether extracellular AREG promotes tissue restoration and survival in sepsis through macrophage pyroptosis. We found that *Areg, Egfr, Il1b, and Il18* were highly expressed in monocytes of patients with severe sepsis compared with those in patients with general sepsis or healthy controls (**Figure 9J**). These findings support the role of extracellular AREG in sepsis pathogenesis.

## 4 Discussion

Growing evidence indicates the employment of LPS as an endotoxin model to explore the mechanisms of inflammatory response in various diseases, such as acute liver (35) or lung injury (36), angiocardiopathy (37, 38), and intestinal damage (39, 40). Liang et al. report that LPS-primed BMDM could facilitate inflammation and oxidative stress, accelerating acute lung injury (41). In addition, LPS-primed BMDM serves as an *in vitro* cell model for inducing lung inflammation and injury (42).

Pyroptosis is a gasdermin-mediated form of programmed cell death (42). Although its crucial role in the innate immune defense is well established, the regulation effects and molecular mechanisms of extracellular AREG in pyroptosis remain unclear. In this study, *in vitro* LPS or extracellular AREG-stimulated BMDMs were employed to explore the regulatory mechanism of extracellular AREG in macrophage pyroptosis. Our findings indicate that extracellular AREG exacerbates pyroptosis in LPS-treated macrophages. We found that extracellular AREG, combined with ATP, induces macrophage pyroptosis via the EGFR/TLR4/NFκB signaling pathway.

AREG is initially described as an epithelial cell-derived factor and mainly regulates cell proliferation, differentiation, apoptosis, and autophagy in several diseases (43, 44). In addition, AREG is also expressed on the surface of alveolar and peritoneal macrophages as a type I transmembrane protein precursor (proAREG) (45–47). When the body is stimulated by inflammatory mediators, extracellular AREG is released into the extracellular matrix or binds to EGFR on the surface of neighboring cells, activating the EGFR signaling pathway (48).

As a critical intracellular nuclear transcription factor, NFκB mainly regulates inflammation, immune response, cell death, and so on (49, 50). NFκB is activated by the EGF/EGFR pathway, contributing to inflammation and cancer progression (51–53). Therefore, understanding the causes of NFκB activation in sepsis is crucial. Here, we found that AREG, a member of the EGF family, was increased in LPS-stimulated RAW264.7 cells. Extracellular AREG then induced IκB phosphorylation, leading to NFκB activation in BMDM. This study further showed that inhibiting EGFR phosphorylation and knockout of TLR4 impairs extracellular AREG-induced NFκB activation in BMDM. Additionally, the inhibition of EGFR phosphorylation also downregulates TLR4 expression. We observed a close connection between TLR4 and EGFR in extracellular AREG-induced NFκB activation in BMDMs. To our knowledge, this is the first report showing extracellular AREG-induced NFκB activation through EGFR/TLR4 signaling.

Inhibiting EGFR phosphorylation and knockout of EGFR significantly decreased LPS-induced TLR4 phosphorylation at Y674A and Y680A (54). Tyrosine phosphorylation of TLR4 is essential for downstream signaling, and TLR4 mutants at Y674A and Y680A of the TIR domain suppress LPS-dependent activation of NFκB (55). Therefore, further investigation is needed to determine whether extracellular AREG promotes TLR4 tyrosine phosphorylation through EGFR interaction.

In the process of determining the underlying effect of extracellular AREG on macrophage pyroptosis, we found that extracellular AREG pretreatment remarkably enhanced LPS+ATP-induced NFκB activation and pyroptosis. This indicates that extracellular AREG promotes macrophage pyroptosis, most likely through NFκB activation. The function of extracellular AREG in pyroptosis differs from other EGFR ligands, possibly because EGFR exerts different cellular responses through direct binding of its ligand, depending on its specific ligand, cell type, or pathological condition (56). Compared with other EGFR ligands, AREG has a low affinity for EGFR, allowing sustained downstream signaling instead of triggering receptor internalization, degradation, and negative feedback loops (57). Thus, we speculated that extracellular AREG persistently activates NFκB signaling, exacerbating macrophage pyroptosis.

The effects of AREG on cytokine production have been studied progressively. Neither exogenous recombinant AREG nor blocking endogenously secreted AREG affects TNF-α, IL-6, and GM-CSF expression in classically activated macrophages (58). Conversely, some studies report that AREG plays a pro-inflammatory role by mediating cytokine production, including IL-6, IL-8, and GM-CSF in epithelial cells (59, 60). In this study, extracellular AREG alone activated the initiation step, including the transcription of *Nlrp3, Caspase1*, *and IL-1b*. The addition of ATP as a second signal further promotes NLRP3 inflammasome assembly, resulting in CASPASE-1 activation and GSDMD cleavage. Overall, AREG is a potential target for regulating macrophage pyroptosis.

AREG signaling, determined via protein processing and trafficking, can be triggered through autocrine, juxtacrine, and paracrine pathways, as well as intracellular nuclear translocation and exosome inclusion (61–63). Extracellular AREG exerts its biological effects through EGFR-mediated intracellular signaling pathways, including Ras/MAPK, PI3K/AKT, mTOR, STAT, and PLCγ. These pathways regulate gene expression and drive multiple cellular responses such as survival, proliferation, angiogenesis, motility, and invasiveness (64–66). AREG plays an important role in LPS-induced macrophage activation (37). However, the effect and mechanism of AREG in LPS-induced macrophage pyroptosis remain unclear. Overall, our findings suggest that extracellular AREG induced macrophage pyroptosis through the EGFR/TLR4/Myd88/NFκB axis. Our study showed an association between serum AREG levels, CRP levels, and the mortality and severity of patients with sepsis. These findings align with that of a previous study, showing that the expression of serum AREG correlates with disease severity in patients with pulmonary fibrosis (67). Most patients with severe COVID-19 (78%) met the sepsis 3.0 criteria, with sepsis-induced acute respiratory distress syndrome (ARDS) being the most common organ dysfunction (88%) (68). Peripheral blood monocyte pyroptosis increases in patients with sepsis and correlates with mortality (69). Monocytes, bone marrow-derived phagocytes, are recruited and differentiate into macrophages in response to bacterial or viral detection. This aids pathogen clearance and inflammation resolution (70). Single-cell transcriptome analysis of monocytes from patients with COVID-19 revealed a subset with high expression of *Areg* and IL-18 related to pyroptosis and enriched EGFR signaling pathway, specifically present in severe sepsis cases (71). In addition, single-cell transcriptome analysis of antigen-presenting cells (including monocytes and a few dendritic cells) from patients with COVID-19 also revealed elevated *Areg* and IL1β expression, associated with pyroptosis in patients with severe sepsis compared with those with mild or moderate sepsis (72). These studies further support our finding that genes of the Areg-mediated pyroptosis signaling pathway were highly expressed in patients with severe sepsis compared with those with mild or moderate sepsis. Although this cohort study is limited to a relatively small patient sample (n = 54) and cannot draw robust conclusions about the role of AREG in sepsis severity and mortality, it holds scientific value and clinical significance.

**Figure 10:**
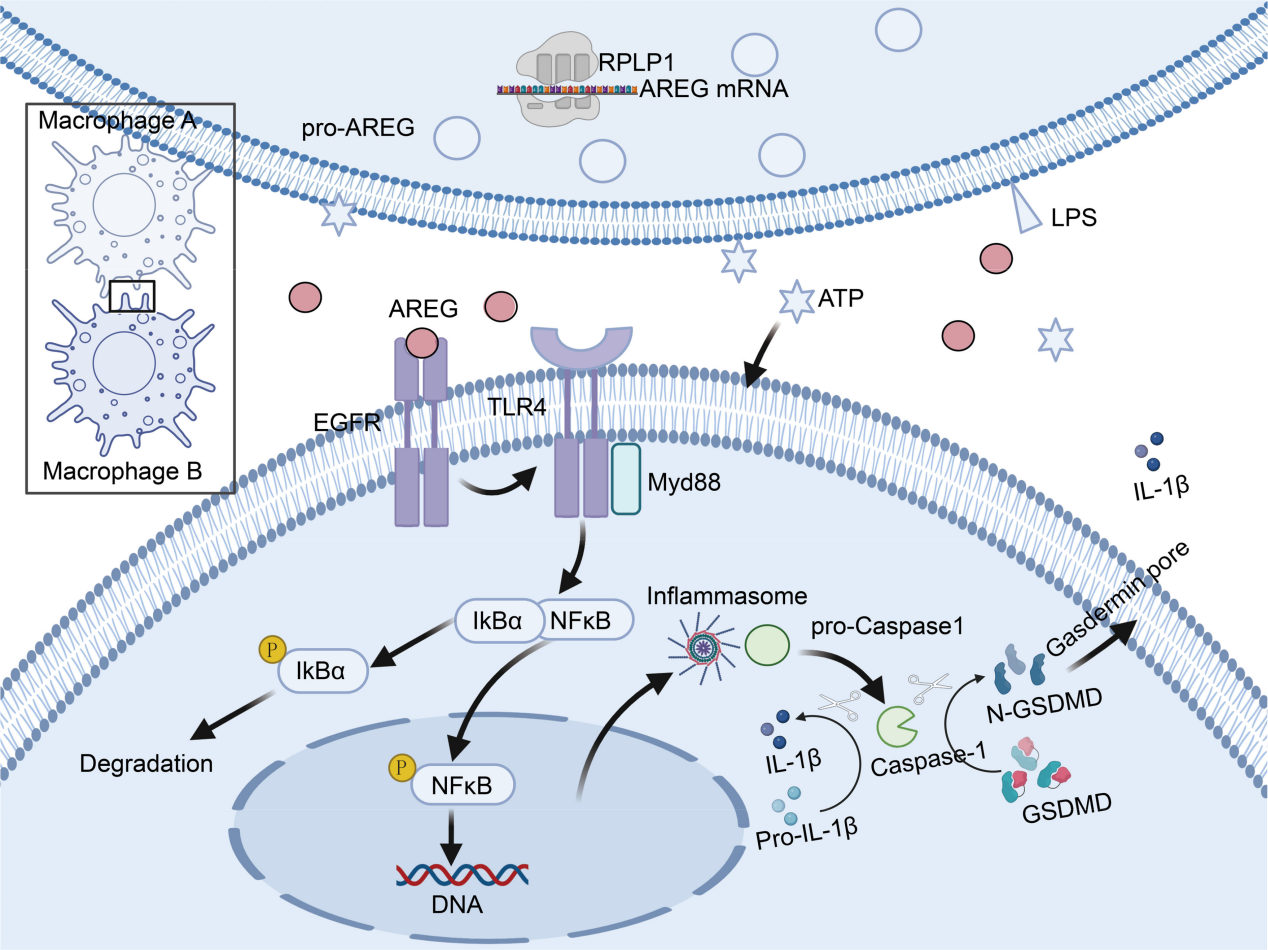
Schematic diagram illustrating how AREG triggers macrophage pyroptosis through the EGFR/TLR4 signaling pathway during inflammatory responses. Schematic diagram was created in https://BioRender.com. Mechanistically, extracellular AREG expression is regulated by the translational regulation of RPLP1. When extracellular AREG and ATP jointly stimulate macrophages, AREG promotes TLR4 expression by binding to EGFR. Expression of TLR4 recruits the adaptor protein Myd88 and further activates downstream IκB and NFκB, which promotes the NLRP3 inflammasome and subsequent pyroptosis. AREG, amphiregulin; EGFR, epidermal growth factor receptor; ATP, adenosine triphosphate.

Our study is the first to show that the molecular mechanisms of extracellular AREG trigger macrophage pyroptosis through the EGFR/TLR4/Myd88/NFκB signaling pathway (**Figure 10**). Additionally, it underscores the potential role of sustained AREG/EGFR signaling in promoting tissue restoration and survival in sepsis. This process may involve the release of inflammatory molecules and metabolites during AREG-induced macrophage pyroptosis, offering a potential treatment strategy for patients with sepsis and ARDS while providing new insights into the pathogenesis of sepsis combined with ARDS.

## Data Availability

The original contributions presented in the study are included in the article/supplementary material. Further inquiries can be directed to the corresponding author.
